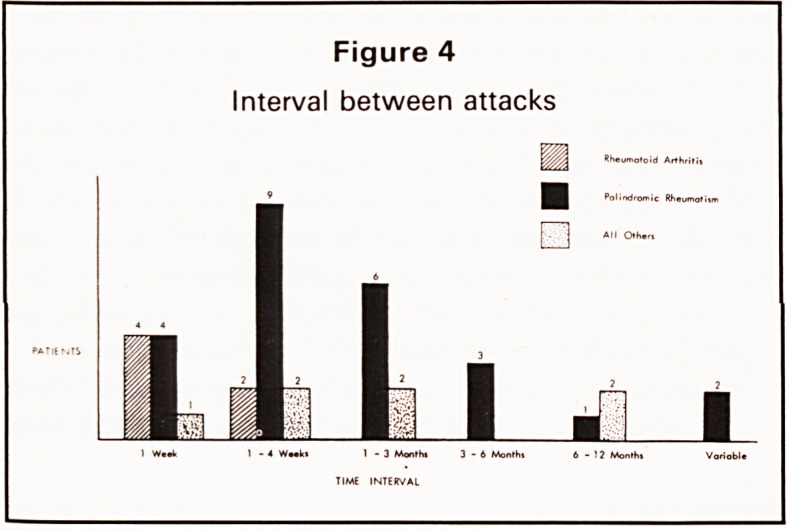# Prognostic Factors in Palindromic Rheumatism

**Published:** 1984-04

**Authors:** C. E. H. Grattan, T. D. Kennedy, D. B. Yates

**Affiliations:** Presently Registrar in Dermatology, Bristol Royal Infirmary, Bristol; Presently Registrar in Medicine, Charing Cross Hospital, London; Consultant Rheumatologist, Musgrove Park Hospital, Taunton

## Abstract

Thirty-eight patients with palindromic rheumatism were reviewed. A questionnaire was compiled to elucidate prognostic factors in the pattern of disease. Over the period of follow-up 6 patients had developed rheumatoid arthritis, 25 remained palindromic, 3 were in remission of symptoms for over a year, 1 developed ankylosing spondylitis, 1 systemic lupus erythematosus and 2 a non-specific polyarthritis. The duration and interval between attacks was variable and did not differ significantly between the palindromic and rheumatoid groups. All patients who developed rheumatoid had a combination of morning stiffness and pain in several joints at once, whereas only 28% of the palindromics did so, (p <0.01). There was a tendency for episodes of joint pain to occur with increasing frequency and for the plasma viscosity to be persistently elevated in those who developed rheumatoid arthritis. Neither a family history of rheumatoid arthritis nor a positive serum rheumatoid factor test at presentation were of prognostic significance. Oral analgesics or symptomatic measures for the relief of joint pain were effective in the majority of patients.


### Prognostic Factors in Palindromic Rheumatism

**C. E. H. Grattan, M.R.C.P.**
Presently Registrar in Dermatology, Bristol Royal Infirmary, Bristol
**T. D. Kennedy, M.R.C.P.**
Presently Registrar in Medicine, Charing Cross Hospital, London
**D. B. Yates, M.R.C.P.**
Consultant Rheumatologist, Musgrove Park Hospital, Taunton

### ABSTRACT

Thirty-eight patients with palindromic rheumatism were reviewed. A questionnaire was compiled to elucidate prognostic factors in the pattern of disease. Over the period of follow-up 6 patients had developed rheumatoid arthritis, 25 remained palindromic, 3 were in remission of symptoms for over a year, 1 developed ankylosing spondylitis, 1 systemic lupus erythematosus and 2 a non-specific polyarthritis. The duration and interval between attacks was variable and did not differ significantly between the palindromic and rheumatoid groups. All patients who developed rheumatoid had a combination of morning stiffness and pain in several joints at once, whereas only 28% of the palindromics did so, ($p < 0.01$). There was a tendency for episodes of joint pain to occur with increasing frequency and for the plasma viscosity to be persistently elevated in those who developed rheumatoid arthritis. Neither a family history of rheumatoid arthritis nor a positive serum rheumatoid factor test at presentation were of prognostic significance. Oral analgesics or symptomatic measures for the relief of joint pain were effective in the majority of patients.

### INTRODUCTION

The diagnosis of palindromic rheumatism (PR) is based on the clinical history, the features of which were first described by Hench and Rosenberg[1] in 1941. It is usually considered a syndrome which may either be the initial manifestation of many different organic processes or a syndrome which does not evolve further. Palindromic simply means 'to recur' or 'to return' and is derived from the Greek palindromo= 'I run back again'. Hench and Rosenberg proposed 'Palindromic Rheumatism' as a descriptive rather than an aetiological term for the recurring and retreating nature of the disease.

The attacks of joint pain, swelling or redness usually last from a few hours to three days although, less commonly, they may persist for up to a week. Typically they appear to flit from joint to joint. Physical examination between attacks is characteristically normal. The erythrocyte sedimentation rate (ESR) between attacks is usually normal but may be raised during attacks.[1] Rheumatoid factor may or may not be present but usually appears coincidently with the development of a chronic polyarthritis.[2] Previous studies indicate that between one third and one half of these cases develop a chronic polyarthritis indistinguishable from rheumatoid arthritis (RA) after a variable interval from months to years.[2-4] The purpose of this study was to identify prognostic factors in the pattern of disease.

### PATIENTS AND METHOD

Fifty patients with a clinical diagnosis of palindromic rheumatism (PR) have been reviewed. They had presented to Rheumatology Clinics in the West Somerset catchment area over five years from 1976 to 1981.

A questionnaire was sent to each patient and of the 43 who replied, 20 were seen in research clinics. Eighteen of the remaining 23 had been seen with the previous 2 years and were included in the analysis. The following questions were asked:

(1) When was your last attack of joint pain or swelling?
(2) How long does a typical attack last? (Less than 1 day, 1–3 days, 3–7 days, more than 7 days.)
(3) How long is there between attacks? (Less than 1 week, 1 week–1 month, 1–3 months, 3–6 months, 6 months to 1 year.)
(4) Do the attacks of joint pain
  (a) involve several joints at once?
  or
  (b) flit from joint to joint?
(5) Do any of your joints feel stiff every morning?

(6) Have any of your relatives had
   (a) a pattern of joint pain like yours?
   (b) rheumatoid arthritis?
   (c) any other form of arthritis?
(7) Which form of treatment do you find most effective in relieving an attack?

At follow-up the questionnaire was reviewed with the patient. A physical examination was performed by one of three physicians. Blood was taken for IgM rheumatoid factor[5] (RF), full blood count (FBC) and plasma viscosity (PV). Hands were x-rayed, together with any other appropriate joint. The films were reviewed independently by a consultant radiologist. Information on the other 18 patients was obtained from the medical records.

On the basis of the results, patients were divided into one of four groups:
(1) Converted to RA (fulfilled 5 or more criteria of the American Rheumatoid Association).[6]
(2) Continued to experience attacks of PR.
(3) Remission of symptoms for over one year.
(4) An alternative diagnosis established.

### RESULTS

The age range of onset was from 16 to 62 years with a peak incidence in the fourth decade (Figure 1). There were 21 females and 17 males. Disease duration ranged from 1 to 15 years, median 4 years (Figure 2). Six patients converted to RA, 25 remained palindromic, 3 were in remission of symptoms for over 1 year, 1 developed ankylosing spondylitis, 1 systemic lupus erythematosus and 2 a non-specific polyarthritis over the period of follow-up (range, 1 month to 15 years, median 3 years).

**Figure 1**
Age of onset

**Figure 2**
Disease duration

### MORNING STIFFNESS AND SIMULTANEOUS POLYARTHRITIS (Table 1)

Patients who experienced pain in several joints at the same time were said to have a simultaneous polyarthritis. Seven of the palindromic group (28%) and 6 of the rheumatoid (100%) developed morning stiffness in combination with a simultaneous polyarthritis ($p<0.01$, $\chi^2$ with Yates's correction).

### ATTACK LENGTH AND INTERVAL BETWEEN ATTACKS (Figures 3 and 4)

There was no significant difference between the length of attack or the interval between attacks in the patients who subsequently converted to RA (group 1) or those who continued to experience PR (group 2). There was a tendency for episodes of joint pain to occur with increasing frequency in those patients who subsequently fulfilled criteria for the diagnosis of RA.

### FAMILY HISTORY (Table 2)

Twenty-three patients gave a family history of joint disease and 14 specified RA. Again there was no significant difference between groups 1 and 2. However 2 patients with PR possessed a first degree relative with a similar pattern of palindromic arthralgia.

### RHEUMATOID FACTOR

A positive latex screening test or sheep cell agglutination titre of 1:32 or more was present in 12/38 patients at presentation. Only 2 of these developed RA. Of the 9 seropositive patients who remained palindromic, 8 were associated with a flitting pattern of joint pain and 4 developed morning stiffness. Four of the 9 became seronegative over the period of follow-up. No seronegative patient who remained palindromic developed a positive rheumatoid factor.

**Table 1**

**Patients with morning stiffness, simultaneous polyarthritis or the combination**

| | *Morning stiffness* | *Simultaneous polyarthritis* | *Combination* | *No combination* |
|---|---|---|---|---|
| RA | 6 | 6 | 6 | 0 |
| PR | 13 | 12 | 7 | 18 |
| | | | $\chi^2 = 7.56$ with Yates's correction, df 1, $p < 0.01$ | |
| Remission | 1 | 2 | 1 | 2 |
| Other diagnosis | 2 | 3 | 1 | 3 |

**Figure 3**

Length of attack

**Table 2**

**Patients with family history of joint disease**

| | *FH of PR* | *FH of RA* | *FH of other joint disease* |
|---|---|---|---|
| RA | 0 | 4 | 0 |
| PR | 2 | 9 | 8 |
| | | $\chi^2 = 1.37$ with Yates's correction, df 1, p = NS | |
| Remission | 0 | 1 | 0 |
| Other | 0 | 0 | 1 |

**Figure 4**

Interval between attacks

**Table 3**

**Most effective treatment**

| | *Oral analgesics* | *Symptomatic measures* | *Nothing* |
|---|---|---|---|
| RA | 3 | 2 | 1 |
| PR | 19 | 4 | 2 |
| Remission | 2 | 0 | 0 |
| Other diagnosis | 2 | 2 | 0 |

**PLASMA VISCOSITY**

At presentation the plasma viscosity was elevated above normal in 19/32, range 1.74–2.06 centipoises (normal range 1.50–1.72 cp). At follow-up, it was elevated in 4/6 of the rheumatoids (normal in one patient on gold therapy, not measured in another), 6/21 of the palindromics (not measured in 4 patients) and in 1/2 of the remitters.

**ORAL ANALGESICS**

Non-steroidal anti-inflammatory drugs and simple analgesics were said to be effective in 68%, symptomatic measures were found to be helpful in 21% and no effective relief could be obtained in a minority (Table 3).

### DISCUSSION

The age distribution of onset and sex ratio are consistent with previous surveys. Dunn[2] noted a peak incidence of onset in the fifth decade. The prevalence of PR in the West Somerset catchment area is approximately 1 : 10,000. As only about 500 cases have been reported in the literature[7] it is probable that the frequency of diagnosis relates to awareness of the condition. With a small population sample statistical comparison on the data can only be meaningful when the observed differences are large. Nevertheless several trends are apparent from this survey.

The most clear-cut prognostic factor to emerge was the combination of morning stiffness with a simultaneous polyarthritis. The development of this combination in patients who remain palindromic may herald the onset of RA. Surprisingly, perhaps, a positive rheumatoid factor was found in almost a third of the patients at presentation but did not appear to influence the prognosis, as only 2 patients developed RA and 4 became seronegative over the course of follow-up. A family history of joint disease was common, especially for RA, but this did not have prognostic significance.

Plasma viscosity is measured routinely in place of ESR in West Somerset and was found to be elevated in 60% of the patients at presentation. A persistently raised plasma viscosity appears to be more common in those who subsequently develop RA. Further studies with larger numbers are needed to show whether an elevated PV is related to a poor prognosis.

Most patients found that non-steroidal anti-inflammatory and simple analgesics were helpful during attacks. Preparations containing aspirin or paracetamol were most often noted to be effective (possibly as a result of prescribing habits). Heat, rest and support alone were effective in some, while others were unable to find any form of relief.

The low proportion of patients in our series who have so far developed RA may be a reflection of the disease duration in our sample, median 4 years. The average duration of disease in Dunn's series was 12.5 years, range 1–30 years.

In conclusion, it appears that there is no certain way of knowing which patients with PR will develop RA but those who develop morning stiffness with a simultaneous polyarthritis in the presence of a raised PV and shortening intervals between attacks are likely to have a poor prognosis.

Correspondence should be addressed to C.E.H.G., Department of Dermatology, Bristol Royal Infirmary, Marlborough Street, Bristol BS2 8HW.

### REFERENCES

1. HENCH, P. S. and ROSENBERG E. F. (1941) Palindromic rheumatism: A 'new' oft-recurring disease of joints (arthritis, peri-arthritis, para-arthritis) apparently producing no articular residues: report of 34 cases. *Staff Meetings of the Mayo Clinic* **16**, 808–815.
2. DUNN, E. C., JONES, D. W., MATTINGLY S., ROBINSON W. M. and WILLIAMS R. A. (1981) The prognosis in palindromic rheumatism. *Ann. Rheum. Dis.* **40**, 206–207.
3. ANSELL, B. M. and BYWATERS E. G. L. (1959) Palindromic rheumatism. *Ann. Rheum. Dis.* **18**, 331–332.
4. WAJED, M. A., BROWN, D. L. and CURREY, H. L. F. (1977) Palindromic rheumatism. Clinical and serum complement study. *Ann. Rheum. Dis.* **36**, 56–61.
5. ROSE, M. H., RAGAN. C., PEARCE, E. and LIPMAN, M. O. (1948) Differential agglutination of normal and sensitised sheep erythrocytes by sera of patients with rheumatoid arthritis. *Proc. Soc. Exp. Biol.* (N.Y.) **68**, 1–6.
6. ROPES, M. W., BENNETT, G. A., COBB, J., JACOX, R. and JESSAR, R. A. (1958) 1958 revision of diagnostic criteria for rheumatoid arthritis. *Bull. Rheum. Dis.* **9**, 175–176.
7. HARDO H. G. (1981) Palindromic rheumatism: a review. *J. Roy. Soc. Med.* **74**, 521–524.

## Figures and Tables

**Figure 1 f1:**
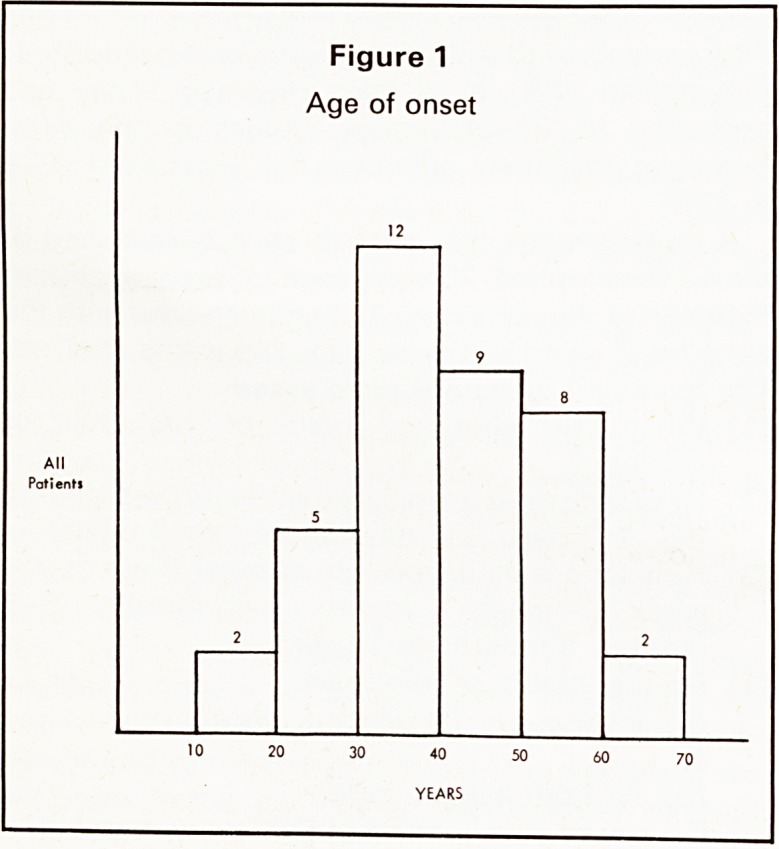


**Figure 2 f2:**
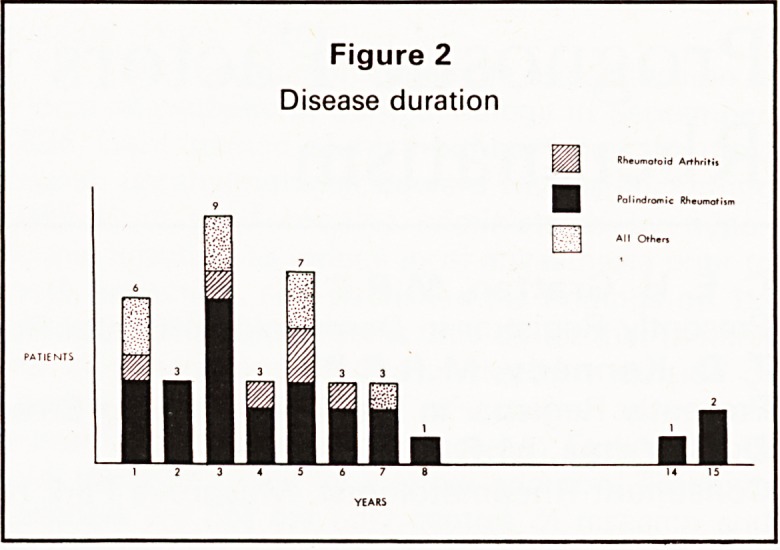


**Figure 3 f3:**
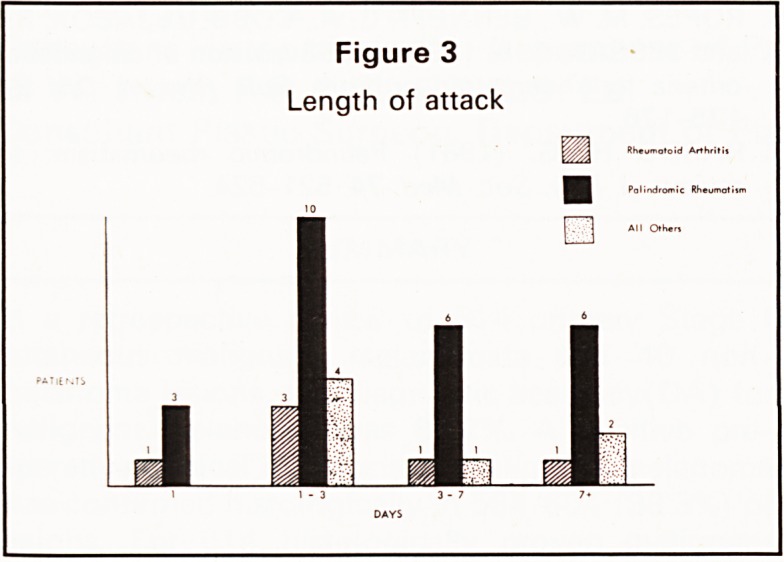


**Figure 4 f4:**